# Prevalence and influence factors of suicidal ideation among females and males in Northwestern urban China: a population-based epidemiological study

**DOI:** 10.1186/s12889-015-2257-5

**Published:** 2015-09-25

**Authors:** Huiwen Xu, Weijun Zhang, Xiaohua Wang, Jiaqi Yuan, Xinfeng Tang, Yi Yin, Shengfa Zhang, Huixuan Zhou, Zhiyong Qu, Donghua Tian

**Affiliations:** School of Social Development and Public Policy, China Institute of Health, Beijing Normal University, 19, Xinjiekou Wai Street, Beijing, 100875 China; Department of Public Health Sciences, University of Rochester School of Medicine and Dentistry, Rochester, NY 14642 USA

**Keywords:** Suicidal ideation, CES-D, Gender differences, Undeveloped urban districts, Northwestern China

## Abstract

**Background:**

Suicide is an urgent public health challenge for China. This study aims to examine the prevalence, influence factors, and gender differences of suicidal ideation among general population in Northwestern Urban China.

**Methods:**

Data used in this study were derived from the third wave of a cohort study of a randomized community sample with 4291 participants (≥20 years) in 2008 in Lanzhou City and Baiyin City, Gansu Province. Data were collected via face-to-face interview by the trained interviewers. Descriptive analyses, chi-square tests and multivariate logistic regressions were performed by using Stata 12.0, as needed.

**Results:**

The prevalence of 12-month suicidal ideation was 4.29 %, there was no significant difference between males and females [5.04 % *vs* 3.62 %, Adjusted Odds Ratio (AOR) = 0.83, *p =* 0.351]. Several risk factors for suicidal ideation were confirmed, including being unmarried (AOR = 1.55, *p* = 0.030), having depression symptoms (AOR = 2.33, *p* < 0.001), having other insurance (AOR = 1.83, *p* = 0.01) or no insurance (AOR = 1.73, *p* = 0.024). In addition, several influence factors were significantly different in males and females, such as being currently married (unmarried *vs* married, AOR = 1.84, *p =* 0.027, for females; no difference for males), feeling hopeless (hopless *vs* hopeful, AOR = 1.92, *p =* 0.06, for females; no difference for males), having other insurances (having other insurances *vs* having basic employee medical insurance, AOR = 1.92, *p =* 0.044, for males; no difference for females), having debts (having debts *vs* no debts, AOR = 2.69, *p =* 0.001, for males; no difference for females), currently smoking (smoking *vs* nonsmoking, AOR = 3.01, *p* = 0.019 for females, no difference for males), and currently drinking (drinking *vs* nondrinking, AOR = 2.01, *p =* 0.022, for males; no difference for females).

**Discussion and conclusion:**

These findings suggested that comprehensive suicide prevention strategies should be developed or strengthened in order to prevent suicide ideation in China, and the gender-specific differences need to be explored through further researches.

## Background

Suicide is an urgent public health challenge which has received the increasing attention in China and worldwide [1, 2]. World Health Organization (WHO) estimated that there were about 1 million people died by suicide, which accounted for 1.3 % of the total global burden of disease in 2004 [3]. In China, suicide was the fifth leading cause of death in China and contributed to 42 % of all suicide worldwide and 56 % of the world’s female suicide [4]. It is worth noting that there are several specific characteristics of suicide in China, including high suicide rate among females [4, 5], high rural suicide rate [5, 6], pesticides poisoning being regarded as a major suicide method [2, 7], and low-planned suicides [8].

In China, higher employment rates and better educational opportunities from recent economic developments may have contributed to the decrease of overall suicide rate [9]. Previous studies showed that the suicide rate had dropped from 22.9 to 15.4 per 100,000 during the period of 1991–2000 [10] and further decreased to 5.28 per 100,000 (5.78 and 4.77 for urban males and urban females respectively; 9.95 and 8.58 for rural males and rural females respectively) in 2011 [11, 12]. However, suicide rates among young males and rural older adults increased recently [12–14]. Taking the 1.4 billion population of China into consideration, the burden of suicide is extremely high [15]. Therefore, suicide is still a major public health challenge in China [3], and more attention should be paid to suicide prevention in the future.

To prevent suicide in advance, suicidal ideation, defined as the wishes to be dead or thoughts of killing oneself, has been introduced by some researchers [16, 17]. Suicidal ideation, which involves a hierarchy of feelings from the thought of “Life is not worth living” to more serious articulation of a thought-out plan, is important because most suicides and parasuicides have engaged in suicidal thoughts prior to their acts [18, 19]. Meanwhile, suicidal ideation is clinically important because it enables the measurement of intent [18]. A previous study had confirmed the importance of suicidal ideation and further suggested that continued efforts should be outreached to the untreated individuals with suicidal ideation before the occurrence of attempts [20]. Hence, a deeper understanding of suicidal ideation will be beneficial in the suicide intervention and prevention.

Previous studies have investigated the prevalence of lifetime, 24-month, and 12-month suicidal ideation in China. The lifetime prevalence was 28.1 % among adults aged 20–59 years in Hong Kong (east coast) [21], 18.5 % in the rural areas of Yuncheng City, Shanxi Province (north China) [22], and 18.8 % in the rural areas of Mianyang City, Sichuan Province (southwest China) [23]. The prevalence of 24-month suicidal ideation was 2.12 % among the rural residents in Zhejiang Province (east China) [24]. It was also found that prevalence of suicidal ideation in a 12-month period was 6.7 % in Hong Kong [25], 5.2 % among rural residents aged 16–34 years [23] and 8.8 % in rural older adults [26] in Mianyang City, and 17.25 % among adolescents in Guangzhou (south China) [27].

In addition, a few of influencing factors have been found to be independently associated with suicidal ideation, including depressive symptoms [28, 29], the severity of depressed mood [30], decrease in income [29], unemployment [31, 32], traumatic life events [28], age, marriage and concern by others [30], the balance between life events and social support [30], and a history of adverse childhood experiences [33]. Among the general population of China, the following risk factors for suicide ideation have also been screened out: being female [23, 25, 34], being elderly [35], being unmarried [23, 25], mood disorders [21, 36], being physically unhealthy [37], having debts [38], feeling helplessness [21, 25], drinking [27, 34], and smoking [25]. Apart from above, some studies found a positive association between Body Mass Index (BMI) and suicidal ideation [39], however the results were not consistent [40–43]. Therefore, the potential mechanism of the obesity-suicidal ideation association remains unclear.

In China, existing studies outlined above were mainly conducted in four developed urban areas (Beijing, Shanghai, Guangzhou, and Hong Kong), and three rural districts (Yuncheng, Mianyang and Zhejiang). However there is no study on the prevalence or influence factors of suicidal ideation conducted in undeveloped urban areas, especially in western China. Furthermore, although gender differences in the suicide [44, 45] and suicide attempts [46–48] have been confirmed, the gender-specific influence factors for suicidal ideation have not been examined in general population, with the exception of a study on adolescents in Guangzhou [27]. To fill these gaps in the current research, a population-based study in western urban China was conducted to investigate the prevalence and influence factors of suicidal ideation, and to examine the gender-specific influence factors of suicidal ideation.

## Methods

### Sampling

The data used in this study were derived from the third wave of the Chinese Urban Social Protection Survey [49–51], a cohort study of a randomized community sample in Lanzhou City and Baiying City of Gansu Province (northwestern China), with the cooperation of the Civil Affairs Department of Gansu Province (northwestern China). The baseline survey was completed in 2005, and subsequently two follow-up rounds of data collection were conducted in April 2006 and January 2008, respectively. This study was approved by the Committee of Ethics of School of Social Development and Public Policy (SSDPP) at Beijing Normal University. Gansu Province is a typical low-income region in western China. In 2012, the per capita disposable income of urban and rural residents was 17,237 RMB and 4,495 RMB respectively, both of which ranked in the bottom of the 31 provinces [52]. Lanzhou City, the capital of Gansu Province, is home to a population of 2,063,800, with a per capita disposable income of 18,442 RMB in 2012 [53]. Baiying City, which is 60 km away from Lanzhou, has a population of 714,200, with a per capita disposable income of 18,532 RMB in 2012 [54].

**Fig. 1:**
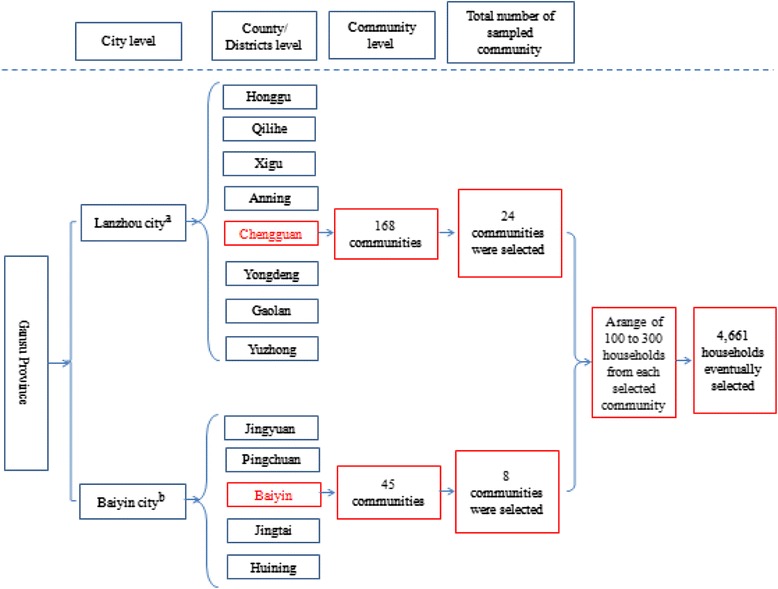
The sampling map in this study

In urban China, there is a four-tier administrative system, including provinces, cities, counties/districts, towns/sub-districts, and communities [23]. Based on the administrative system, a three-stage cluster sampling process was employed, which was showed in Fig. 1. In the first two stages, the “probability proportional to size” (PPS) sampling method was used to select districts and communities based on their population size, and the detailed information can be found in our published articles [49, 50, 55]. During the third stage, 100 households were set to be the equivalent of one sampling unit; the final number of households included in the sample was determined according to the number of sampling units in each community [49]. Thus, using simple random sampling, the final sample includes a range of 100 to 300 households from each selected community. A total of 4,661 households from 32 communities throughout the two cities were eventually selected. In each household, one representative, who aged 20 years and over and was familiar with the familial situation, was responsible for the household survey and received the psychological assessments. All contacted family members provided the information about the social-demographic characteristics, medical service utilization, social protection, and employment status. As a result, the surveyed households and household respondents totaled 4,661 and 13,051, respectively. Of them, 4,291 household representatives, who successfully completed the psychological assessments, were involved in this study, yielding a response rate of 92.1 %. For the remaining household representatives, 201 people refused to participate in this study, 60 people could not been reached for more than three times, and the reminding 109 questionnaires were discarded because of the bad quality.

### Interviewer training, quality control and interview setting

The survey was completed via face-to-face interviews by the trained interviewers. 50 graduate students, with a major in psychology, sociology or social policy, were selected as the interviewers from Northwest Normal University (NNU), Lanzhou City. All the interviewers participated in a 7-day systematic training at NNU, which was taught by experienced researchers from the School of Social Development and Public Policy (SSDPP) of Beijing Normal University (BNU). After finishing the training, interviewers were supposed to acquire following knowledge: (1) the purpose of the survey, (2) the standard interviewing protocol and techniques, and (3) the explanations of the items in the questionnaire. Finally, 40 qualified interviewers were recruited after a face-to-face assessment.

To ensure the quality of survey, a guideline for investigation, which covered detailed information about the research objective, question definitions, and a step-by-step interviewing protocol, was sent to each interviewer. Furthermore, prior to the interview, a letter was delivered to each selected family to inform them of the purpose of the survey and their right to refuse it. Prior to the start of interview, an inform consent document was signed once the respondent agreed to join the survey. The whole interview took about 30 to 60 minutes, which depended on the family size and the understanding capacity of the respondents. The entire survey took 12 days in January 2008. In addition, a quality control group that included five experienced researchers from SSDPP were responsible for monitoring the process of interview, and checking the questionnaires at the end of each working-day. The repeated interview was conducted through the telephone when over 30 % of data were missed in a questionnaire. Eventually, 120 questionnaires were completed by the supplementing interviews.

### Measures

The major questions about the variables used in this study are described in Table 1. Participants were asked the following question about general suicidal ideation: “During the past 12 months, have you ever considered suicide?” (yes/no) [23, 56]. Depression status in this study was measured using the 20-item Center for Epidemiologic Studies Depression Scale (CES-D) Chinese version [57], with a score of 21 serving as the cut-off point for moderate to severe depression status [58, 59], and the Cronbach’s Alpha of 0.87 for 20 items. The data processing methods has been explicated in our previous articles [49, 50, 55]. In brief, social-demographic characteristics included gender (male, female), age (20–34, 35–44, 45–54, 55–64, 65 plus), education (junior high school or less, high school, college or above), currently married (yes, no). BMI was calculated by weight in kilograms divided by height in meters squared (Kg/m^2^), and recoded as a three-category variable (<19, ≥19& < 25, ≥25). The self-rated health was an ordinal five-point variable, ranked as very good, good, fair, bad, and very bad. Responses of “very good” and “good” were collapsed into the “good” category, and responses of “poor” and “very poor” were recoded as “poor”. Additionally, the hope for the future was categorized as very hopeful or hopeful, fair, hopeless or very hopeless, and unknown. Based on the health insurance structure in urban China [60], the variable of health insurance included the following categories: Basic Employee Medical Insurance (BEMI), Government Insurance Scheme, Medical Aid, private medical insurance, other programs related to medical services, and uninsured. As the BEMI is the basic health insurance provided for urban employees in China, and covers both the outpatient and inpatient services [60], so the variable was recoded as BEMI, other insurances, and uninsured. The household annual expenditure was derived from the summation of the 16 items listed in Table 1, and was categorized into three groups: ≤10,000 RMB, >10,000 & ≤ 20,000 RMB, and >20,000 RMB. Debts, smoking status and drinking status were all dichotomized variables (yes/no); employment status was grouped into employed, retired, and unemployed.

**Table 1:** Wording of questions comprising variables included in the analyses

Variables	Question
Suicidal ideation	During the past 12 months, did you ever consider suicide?
Currently married	What is your current marital status?
Body mass index	What is your height in centimeter? & what is your weight in kilogram?
Self-rated health	How would you rate your health when compared with same-aged peers?
Depression	Center for Epidemiologic Studies Depression Scale
Hope	Do you feel hopeful about the future?
Health insurance	What is your health insurance?
Household annual expenditure (RMB/year)	What is the annual household expenditure for the following items: food, smoke& alcohol, clothes, daily necessities, durable goods, labor services, children education, adult education and training, alimony payment, gifts, transportation, water/electricity, communication, medical service, house renting, others.
Debts	During the past year, did your family have any debts?
Employment	What is your current employment status?
Currently smoking	During the past month, whether do you smoke or not?
Currently drinking	During the past month, whether do you drink or not?

### Data entry and statistics analysis

Statistical analysis in this study involved in four steps. First, cross-tabulations were computed to estimate the prevalence of suicidal ideation in all of the respondents and among different groups. *P* values were obtained to test the difference between independent variables and suicidal ideation. As suicidal ideation is a dichotomized variable, logistic regression model was used in this study. Univariate logistic regressions were then performed separately for all independent variables. Next, all the procedures above were conducted in the female and male subgroups. Then all variables used in the univariate analysis entered into a multivariate logistic regression simultaneously. The adjusted odd ratios (AORs), 95 % confidence intervals (95 % CI) and *P* values of influence factors were obtained. Finally, two separate logistic regressions were performed to determine associations between influence factors and suicidal ideation among males and females, respectively. In addition, the listwise deletion was employed to address the missing data in all statistical analyses. The level of significance was set as 5 % throughout the study. Data analyses were performed using Stata12.0 (StataCorp LP, College Station, Texas, USA).

## Results

Of the 4291 respondents in this study, 184 (4.29 %) reported having suicidal ideation during the past 12 months. The distributions of suicidal ideation among different influence factors and the results of univariate logistic analysis were shown in Table 2. Specifically, males reported less suicidal ideation than females (3.62 % vs. 5.04 %). Furthermore, the univariate analysis showed that (Table 2) there were significantly statistical difference among all the independent variables (all had a *p* value < 0.05), except for the following variables: age (*p* = 0.966), education (*p* = 0.138), smoking (*p* = 0.719), and drinking (*p* = 0.126). Participants, who were unmarried, had abnormal BMI, reported self-rated poor health, were depressed, felt hopeless for the future, did not have insurance or other insurances, had lower family expenditure, had debts, and were unemployed reported suicidal ideation more often than their counterparts (all *p value* < 0.05).

**Table 2:** Prevalence and univariate analysis of influence factors of suicidal ideation

Variables	N	Non-suicidal ideation, n (%)	Suicidal ideation, n (%)	*p*	OR (95 % CI)
All respondents	4291	4107 (95.71)	184 (4.29)		
Gender					
Male	2349	2264 (96.38)	77 (3.62)	0.024	0.71 (0.52–0.95)^a^
Female	1846	1753 (94.96)	93 (5.04)		1 (referent)
Age					
20–34	330	318 (96.6)	12 (3.64)	0.966	1 (referent)
35–44	998	954 (95.59)	44 (4.41)		1.22 (0.64–2.34)
45–54	1013	967 (95.46)	46 (4.54)		1.26 (0.66–2.41)
55–64	693	664 (95.82)	29 (4.18)		1.16 (0.58–2.30)
≥65	1257	1204 (95.78)	53 (4.22)		1.17 (0.62–2.21)
Education					
Junior high school or less	2440	2324 (95.25)	116 (4.75)	0.138	1 (referent)
High school	1385	1334 (96.32)	51 (3.68)		0.77 (0.55–1.07)
College or above	336	326 (97.02)	10 (2.98)		0.61 (0.32–1.18)
Currently married					
No	904	840 (92.92)	64 (7.08)	<0.001	2.10 (1.54–2.88)^a^
Yes	3291	3176 (96.51)	115 (3.49)		1 (referent)
BMI					
<19	279	259 (92.83)	20 (7.17)	0.011	1.92 (1.18–3.12)^a^
≥19 & <25	3464	3330(96.13)	134(3.87)		1 (referent)
≥25	548	518 (94.53)	30 (5.47)		1.44 (0.96–2.16)
Self-rated health					
Good	1833	1771 (96.62)	62 (3.38)	<0.001	1 (referent)
Fair	1890	1813 (95.93)	77 (4.07)		1.21 (0.86–1.71)
Bad	480	441 (91.88)	39 (8.13)		2.53 (1.67–3.82)^a^
CES-D Scores					
≥21	1315	1209 (91.94)	106 (8.06)	<0.001	3.51 (2.58–4.78)^a^
<21	2914	2843 (97.56)	71 (2.44)		1 (referent)
Hope					
Hopeful	1866	1810 (97.00)	56 (3.00)	<0.001	1 (referent)
Fair	943	905 (95.97)	38 (4.03)		1.36 (0.89–2.06)
Hopeless	513	459 (89.47)	54 (10.53)		3.80 (2.58–5.60)^a^
Unknown	960	925 (96.35)	35 (3.65)		1.22 (0.80–1.88)
Health Insurance					
BEMI	1948	1900 (97.54)	48 (2.46)	<0.001	1 (referent)
Other insurances	897	838 (93.42)	59 (6.58)		2.79 (1.89–4.11)^a^
Uninsured	1137	1074 (94.46)	63 (5.54)		2.32 (1.58–3.41)^a^
HAE					
≤10000	1825	1723 (94.41)	102 (5.59)	<0.001	1 (referent)
>1000& ≤ 20000	1479	1437 (97.16)	42 (2.84)		0.49 (0.34–0.71)^a^
>20000	987	947 (95.95)	40 (4.05)		0.71 (0.49-1.04)
Debts					
Yes	3597	3474 (95.68)	123 (3.42)	<0.001	2.72 (1.98–3.74)^a^
No	694	633 (91.21)	61 (8.79)		1 (referent)
Employment					
Employed	1219	1179 (96.72)	40 (3.28)	0.001	1 (referent)
Retired	1749	1684 (96.28)	65 (3.72)		1.14 (0.76–1.70)
Unemployed	1235	1161 (94.01)	74 (5.99)		1.88 (1.27–2.78)^a^
Currently smoking					
Yes	1077	1030 (95.64)	47 (4.36)	0.719	1.07 (0.72–1.50)
No	2946	2825 (95.89)	121 (4.11)		1 (referent)
Currently drinking					
Yes	668	632 (94.61)	36 (5.39)	0.126	1.34 (0.92–1.94)
No	3623	3475 (95.91)	148 (4.09)		1 (referent)

*OR* odds ratio, *CI* confidence interval, *BEMI* Basic Employee Medical Insurance, *HAE* household annual expenditure

*P* values were associated with chi-square tests for comparing suicidal ideation by predictors

^a^Significant ORs

The prevalence of suicidal ideation among the different influence factors for females and males were showed in Table 3. The results of chi-square tests indicated that suicidal ideation among both females and males differed significantly by marital status, self-rated health, depression status, hope, health insurance, and debts (all *p value* <0.05). However, smoking was only found to be significant among females (*p* = 0.005); conversely, BMI (*p* = 0.001), employment (*p* = 0.001), and drinking (*p* = 0.027) were only significantly associated with suicidal ideation among males. Univariate logistic analyses confirmed the trends in the correlation analyses for females and males.

**Table 3:** Prevalence and OR (95 % CI) of suicidal ideation among females and males

	Females			Males				
Variables	N	Ideation, n (%)	P	OR (95 % CI)	N	Ideation, n (%)	P	OR (95 % CI)
Age								
20–34	156	8 (5.13)	0.958	1 (referent)	174	4 (2.30)	0.275	1 (referent)
35–44	437	21 (4.81)		0.93 (0.40–2.15)	554	23 (4.15)		1.84 (0.63–5.40)
45–54	364	16 (4.40)		0.85 (0.36–2.03)	641	30 (4.68)		2.09 (0.73–6.01)
55–64	304	17 (5.59)		1.10 (0.46–2.60)	388	12 (3.09)		1.36 (0.43–4.27)
≥65	585	31 (5.30)		1.04 (0.47–2.30)	592	16 (2.70)		1.18 (0.39–3.58)
Education								
Junior high school	1191	61 (5.12)	0.920	1 (referent)	1237	53 (4.28)	0.131	1 (referent)
High school	515	24 (4.66)		0.91 (0.56–1.47)	864	27 (3.13)		0.72 (0.45–1.16)
College or above	117	6 (5.13)		1.00 (0.42–2.37)	215	4 (1.86)		0.42 (0.15–1.18)
Currently married								
No	591	45 (7.61)	0.001	2.06 (1.35–3.13)^a^	307	17 (5.54)	0.05	1.71 (0.99–2.96)
Yes	1247	48 (3.85)		1 (referent)	2027	67 (3.31)		1 (referent)
BMI								
<19	160	10 (6.25)	0.680	1.26 (0.64–2.49)	117	9 (7.69)	0.001	2.70 (1.31–5.60)^a^
≥19 & <25	1474	74 (5.02)		1 (referent)	1973	59 (2.99)		1 (referent)
≥25	212	9 (4.25)		0.84 (0.41–1.70)	259	17 (6.56)		2.28 (1.30–3.97)^a^
Self-rated health								
Good	742	30 (4.04)	0.051	1 (referent)	1085	32 (2.95)	0.002	1 (referent)
Fair	848	43 (5.07)		1.27 (0.79–2.04)	1030	34 (3.30)		1.12 (0.69–1.83)
Bad	252	20 (7.94)		2.05 (1.14–3.67)^a^	223	17 (7.62)		2.71(1.48–4.98)^a^
CES-D Scores								
≥21	595	57 (9.58)	<0.001	3.74 (2.41–5.78)^a^	672	44 (6.55)	<0.001	3.03 (1.94–4.74)^a^
<21	1233	34 (2.76)		1 (referent)	1638	37 (2.26)		1 (referent)
Hope								
Hopeful	802	27 (3.37)	<0.001	1 (referent)	1017	28 (2.75)	<0.001	1 (referent)
Fair	405	22 (5.43)		1.65 (0.93–2.93)	521	15 (2.88)		1.05 (0.55–1.98)
Hopeless	230	30 (13.04)		4.31 (2.50–7.41)^a^	262	22 (8.40)		3.24 (1.82–5.76)^a^
Unknown	391	13 (3.22)		0.95 (0.49-1.87)	546	20 (3.66)		1.34 (0.75–2.41)
Health insurance								
BEMI	669	16 (2.39)	<0.001	1 (referent)	1275	32 (2.51)	0.002	1 (referent)
Others	458	32 (6.99)		3.07 (1.66–5.66)^a^	433	26 (6.00)		2.48 (1.46–4.21)^a^
Uninsured	603	39 (6.47)		2.82 (1.56–5.10)^a^	526	24 (4.56)		1.85 (1.08–3.18)^a^
HAE								
≤10000	857	48 (5.60)	0.579	1 (referent)	915	48 (5.25)	0.001	1 (referent)
>1000& ≤ 20000	585	26 (4.44)		0.78 (0.48–1.28)	872	16 (1.83)		0.34 90.19–0.60)^a^
>20000	404	19 (4.70)		0.83 (0.48–1.43)	562	21 (3.73)		0.70 (0.42–1.18)
Debts								
Yes	284	29(10.21)	<0.001	2.66 (1.68–4.21)^a^	369	28 (7.59)	<0.001	2.77 (1.72–4.42)^a^
No	1562	64 (4.10)		1 (referent)	1980	57 (2.88)		1 (referent)
Employment								
Employed	395	17 (4.30)	0.551	1 (referent)	819	23 (2.81)	0.001	1 (referent)
Retired	783	38 (4.85)		1.13 (0.63–2.04)	962	27 (2.81)		1.00 (0.57–1.76)
Unemployed	661	38 (5.75)		1.36 (0.75–2.44)	560	34 (6.07)		2.24 (1.30–3.84)^a^
Currently smoking								
Yes	59	8 (12.31)	0.005	2.87 (1.32–6.23)^a^	1007	39 (3.87)	0.458	1.18 (0.76–1.84)^a^
No	1653	77 (4.66)		1 (referent)	1275	42 (3.29)		1 (referent)
Currently drinking								
Yes	145	8 (5.52)	0.783	1.11 (0.53–2.34)	446	24 (5.38)	0.027	1.72 (1.06–2.79)^a^
No	1701	85 (5.00)		1 (referent)	1903	61 (3.21)		1 (referent)

*OR* odds ratio, *CI* confidence interval, *BEMI* Basic Employee Medical Insurance, *HAE* household annual expenditure

*P* values were associated with chi-square tests for comparing suicidal ideation by predictors

^a^Significant ORs

The results of multivariate logistic regression model were showed in Table 4. In the full model, being currently unmarried (AOR = 1.55, *p* = 0.030), depression (AOR = 2.33, *p* < 0.001), feeling hopelessness (AOR = 1.78, *p* = 0.001), having other insurances (AOR = 1.83, *p* = 0.011) or no insurance (AOR = 1.73, *p* = 0.024), having debts (AOR = 2.05, *p* < 0.001), and currently drinking (AOR = 1.83, *p* = 0.016) were positively associated with suicidal ideation. For females, being currently unmarried (AOR = 1.84, *p* = 0.027), depression (AOR = 2.79, *p* < 0.001), feeling hopeless (AOR = 1.92, *p* = 0.060), having debts (AOR = 2.69, *p* < 0.001), and currently smoking (AOR = 3.01, *p* = 0.019) were positively associated with suicidal ideation. Among males, depression (AOR = 1.99, *p* = 0.009), having other insurances (AOR = 1.92, *p* = 0.044), and currently drinking (AOR = 2.01, *p* = 0.022) was positively associated with suicidal ideation. A negative association was also found between household annual expenditure between >10,000& ≤ 20000 RMB and suicidal ideation among males (AOR = 0.52, *p* = 0.050). Only depression was significantly associated with suicidal ideation among both females and males. BMI, self-rated health and employment were not significantly in both genders. It is worth noting that smoking and drinking were not significant in the full model; however, smoking was a risk factor for females, and drinking was a risk factor for males.

**Table 4:** Multivariate logistic regressions of suicide ideation among females and males^a^

	All		Females		Males	
Variables	AOR (95 % CI)	*p*	AOR (95 % CI)	*p*	AOR (95 % CI)	*p*
Gender			-		-	
Male	0.83 (0.55–1.24)	0.351				
Female	1 (referent)					
Age						
20–34	1 (referent)		1 (referent)		1 (referent)	
35–44	1.00 (0.51–2.01)	0.980	0.68 (0.26–1.76)	0.428	1.66 (0.55–5.04)	0.369
45–54	0.87 (0.43–1.77)	0.705	0.50 (0.18–1.42)	0.195	1.49 (0.49–4.55)	0.488
55–64	0.93 (0.41–2.09)	0.857	1.16 (0.37–3.46)	0.794	0.94 (0.25–3.49)	0.921
≥65	0.74 (0.33–1.67)	0.474	0.78 (0.26–2.34)	0.655	0.78 (0.20–3.01)	0.716
Education						
Junior high school or less	1 (referent)		1 (referent)		1 (referent)	
High school	0.78 (0.53–1.17)	0.230	1.10 (0.58–2.10)	0.769	0.73 (0.44–1.24)	0.246
College or above	0.94 (0.46–1.93)	0.881	1.40 (0.50–3.95)	0.522	0.64 (0.22–1.88)	0.418
Currently married						
No^c^	1.55 (1.05–2.29)^b^	0.030	1.84 (1.07–3.15)^b^	0.027	1.28 (0.69–2.35)	0.436
Yes	1 (referent)		1 (referent)		1 (referent)	
BMI						
<19	1.30(0.72–2.32)	0.381	0.78 (0.33–1.85)	0.574	2.03 (0.90–4.58)	0.086
≥19 & <25	1 (referent)		1 (referent)		1 (referent)	
≥25	1.09 (0.66–1.80)	0.738	0.67 (0.29–1.56)	0.356	1.64 (0.85–3.15)	0.140
Self-rated health						
Good	1 (referent)		1 (referent)		1 (referent)	
Fair	0.86 (0.58–1.28)	0.458	0.86 (0.48–1.55)	0.622	0.93 (0.54–1.59)	0.781
Bad	1.13 (0.67–1.91)	0.654	1.01 (0.47–2.14)	0.987	1.31 (0.61–2.80)	0.486
CES-D Scores						
≥21	2.33 (1.61–3.37)^b^	<0.001	2.79 (1.61–4.83)^b^	<0.001	1.99 (1.19–3.33)^b^	0.009
<21	1 (referent)		1 (referent)		1 (referent)	
Hope						
Hopeful	1 (referent)		1 (referent)		1 (referent)	
Fair	0.85 (0.53–1.38)	0.722	0.97 (0.50–1.90)	0.928	0.73 (0.35–1.51)	0.399
Hopeless^c^	1.78 (1.12–2.85)^b^	0.001	1.92 (0.97–3.79)	0.060	1.68 (0.86–3.29)	0.128
Unknown	0.99 (0.61–1.62)	0.595	0.64 (0.29–1.42)	0.270	1.35 (0.72–2.56)	0.352
Health Insurance						
BEMI	1 (referent)		1 (referent)		1 (referent)	
Other insurances^c^	1.83 (1.15–2.92)^b^	0.011	1.81 (0.88–3.75)	0.107	1.92 (1.02–3.64)^b^	0.044
Uninsured	1.73 (1.07–2.78)^b^	0.024	2.00 (0.99–4.03)	0.054	1.49 (0.74–3.00)	0.270
HAE						
≤10,000	1 (referent)		1 (referent)		1 (referent)	
>10,000& ≤ 20000^c^	0.94 (0.61–1.43)	0.759	1.55 (0.86–2.77)	0.143	0.52 (0.27–1.00)^b^	0.050
>20,000	1.32 (0.85–2.04)	0.217	1.40 (0.71–2.74)	0.328	1.19 (0.66–2.15)	0.554
Debts						
Yes^c^	2.05 (1.38–3.04)^b^	<0.001	2.69 (1.53–4.72)^b^	0.001	1.64 (0.91–2.96)	0.102
No	1 (referent)		1 (referent)		1 (referent)	
Employment						
Employed	1 (referent)		1 (referent)		1 (referent)	
Retired	1.32 (0.73–2.40)	0.355	1.11 (0.46–2.70)	0.811	1.78 (0.74–4.30)	0.199
Unemployed	0.97 (0.58–1.62)	0.915	0.99 (0.46–2.15)	0.979	0.95 (0.46–1.98)	0.900
Currently smoking						
Yes^c^	1.03 (0.64–1.65)	0.899	3.01 (1.20–7.56)^b^	0.019	0.77 (0.45–1.31)	0.338
No	1 (referent)		1 (referent)		1 (referent)	
Currently drinking						
Yes^c^	1.83 (1.12–2.99)^b^	0.016	1.86 (0.71–4.88)	0.205	2.01 (1.11–3.64)^b^	0.022
No	1 (referent)		1 (referent)		1 (referent)	

*AOR* adjusted odds ratio, *CI* confidence interval, *BEMI* Basic Employee Medical Insurance, *HAE* household annual expenditure

^a^Multivariate logistic models were adjusted age and education as categorical variables in all models

^b^Significant AORs

^c^Statistically significant gender-related difference with suicidal ideation among different predictors

## Discussion

### Comparison of prevalence with other studies

The self-reported 12-month prevalence of suicidal ideation was 4.29 % (3.62 % for male, and 5.04 % for female) among urban adults (≥20 years) in northwestern China. The 12-month prevalence in this study was lower than that in rural Chinese aged 16–34 years (5.2 %) [23] and that in older adults aged 50 years or older in rural China (8.8 %) [26]; however, it was higher than that in rural residents from developed Zhejiang province [24]. Theoretically, the lifetime prevalence of suicide ideation was higher than the 12-month prevalence [1]. Therefore, we can conclude that the 12-month prevalence of suicidal ideation in undeveloped urban districts may be higher than that in developed metropolises, and lower than that in undeveloped rural areas. In other words, this study confirmed the protective effects of economic development and urbanization against suicide and suicidal ideation in China [23, 61, 62].

### Influence factors of suicidal ideation

In this study, all influence factors examined in univariate analysis were found to be significantly different in subjects with and without suicide ideation, except for age, education, drinking and smoking (Table 2). Subsequently, multivariate logistic regressions further confirmed several risk factors for suicidal ideation including being unmarried [23, 25], depression [21, 36, 37], feeling hopeless [21, 25], having debts [38, 63] and currently drinking [27, 34]. It is important to point out that there were disparities between results of univariate regression analysis and multivariate regression analysis. These disparities may be led by the difference of the statistical methods, the univariate regression analysis help us investigate whether a relationship exists between two paired variables, however, multivariate regression analysis aims to determine which variables influence the outcome among a set of variables.

It was intriguing to find that BEMI served as a protective factor against suicidal ideation when compared with subjects who were uninsured and those with other insurances. A recent study, conducted by our research team, has also found a protective function of basic health insurance against depression [49], and this study further confirmed the protective impact of basic health insurance on suicidal ideation in the general population. However, the protective role of insurance, after achievement of universal coverage, should be further examined by longitudinal study in the future.

Age was not significantly associated with suicidal ideation, even those aged 65 or older did not show higher prevalence of 12-month suicidal ideation in this study. It was analogous to the situation of finding a similar prevalence among working-age adults and older adults in Hong Kong [21, 37], but was inconsistent to the results of rural residents in Sichuan Province [23, 26]. Even though, we would like to point out that there has been no large-scale study on this topic by using standardized assessment tools among both rural and urban areas in mainland China. Therefore, the epidemiology of suicidal ideation in China needs to be explored through further researches.

Furthermore, education, employment status, and BMI were not significantly associated with suicidal ideation in multivariate regression model. Previous studies, conducted in China [23, 64] and France [65], showed that lower education levels and being unemployed were significantly associated with increased odds of reporting suicidal ideation. However, another study, conducted by Bromet, E. J. et al. [66], showed that low education were not significant risk factors for the lifetime ideation. These discrepancies may be due to the characteristics of sample, and need to be further studied. It is important to point out that the association between BMI and suicidal ideation still remains unclear. Carpenter et al. concluded that increased BMI was associated with higher suicide ideation for women, however, lower BMI was associated with suicide ideation for men [40]. The overall significant positive associations between BMI and suicidal ideation were also found by Mather et al. [41], Wagner et al. [42], and Dutton, G. R. et al. [43]. Among U.S. youth, perceived overweight was a statistically significant risk factor for suicide attempts [67]. However, Goldney, R. D. et al. [68] revealed that the assumption “increased BMI is necessarily associated with suicidal ideation” was not tenable.

### Gender difference of prevalence and risk factors

The higher prevalence of suicidal ideation among females (female *vs*male: 5.04 % *vs* 3.62 %) was consistent with previous findings in Chinese population [4, 23]. However, being female was not a significant variable in the regression model after controlling other variables (AOR = 0.83, *p* = 0.351). The disappearance of gender-difference in suicidal ideation is contrary to previous studies, in which females experienced a significantly greater risk of reporting suicidal ideation than males [23, 26]. A recent meta-analysis also estimated that AOR of the pooled suicidal ideation for females was 1.43 (95 % CI = 1.25-1.64) in a Chinese population [34]. The disappearance of gender-differences in this study may be partly explained by the characteristics of this sample. For example, females in our sample came from urban community, and their social-economic statuses, compared with males, were relatively better than those from rural areas. Actually, it was found that the female–male suicide ratio has decreased sharply [12], because of the improvement in females’ social-economic status in recent years [23]. However, this interpretation need to be further examined in the future.

This study revealed the different influence factors of suicidal ideation between females and males, which were less emphasized by previous studies. The only shared risk factor of suicidal ideation was depression, a well-known risk factor for suicide and suicidal ideation [34], suggests that mental illness is still a major challenge for preventing suicide in China. In this sample, debt was only associated with suicidal ideation in females but not in males. In addition, household annual expenditure of >10,000& ≤ 20,000 RMB was a protective factor against suicidal ideation in males (marginal difference, *p* = 0.05), but not in females. These results indicated that males benefit more from better economic status than females, while females were easier influenced by negative economic situation (i.e., having debts). Females may be more vulnerable when they experienced the life-events including being unmarried, depression, uninsured, and having debts, compared with males. This finding is inconsistent with the results of studies from western countries, which found debts were significantly correlated with higher suicidal ideation among general population. A study [69] in European and American countries found that the 2008 global economic crisis mainly influenced the suicide rate in men. Another study, conducted by Turvey C et al., revealed that financial loss rather than low income remained a significant correlate of suicidal ideation after controlling for depression [29]. Similarly, Hintikka et al. concluded that difficulty in repaying debts is an independent factor that is associated with suicidal ideation [70]. A more recent study also indicated that the population in debt were twice as likely to have suicidal ideation after controlling for the socio-demographic, economic, social and lifestyle factors [63]. Another more recent study also indicated that poorer financial perception was associated with more prevalent suicidal ideation [23]. One possible explanation for the difference may be that the traditional cultural values in China were different from western countries. Of course, this hypothesis should be further explored.

Finally, smoking and drinking status were not significant influence factors of suicidal ideation in the full model; however, smoking was a significant risk factor in females, and drinking was a significant factor in males. This difference may be explained by the much higher smoking rate in males than females in China (52.9 % vs. 2.4 %) [71]. Two studies showed that current smoking were associated with suicidal ideation [25, 27], another two studies further indicated that daily smoking was associated with suicidal ideation in women but not in men [65, 72]. However, there were no significant differences between suicidal ideation and smoking status in other studies [66, 73]. Now, China is the largest consumer of tobacco in the world, with an estimated 301 million current smokers. Therefore, the inter-relationships of smoking and suicide ideation need to be considered in the future. On the other hand, drinking was a risk factor for males in this study, which was similar with previous studies which confirmed alcohol abuse disorders are more common in males suicides [46, 74].

### Limitations and Strengths of this study

Several limitations of this study should be acknowledged. First, as a cross-sectional study, this study was unable to prove a causal-effect relationship between risk factors and suicidal ideation. Second, as the sample was selected from two cities of Gansu Province, a conservative generalization of the findings of this study should be applied when considering the diversity of social-economic status on other populations. Third, suicidal ideation was assessed by using a single question, which could not distinguish the severity of the suicide risk. The independent measuring tools should be utilized in epidemiological or clinical study in the future. Fourth, more valid measures, such as Beek Depression Invertory Beck (BDI-21) and Patient Health Questionnaire (PHQ-9), should be used in the future study, although the CES-D scale has been used extensively in previous studies. Fifth, social support and personality were not measured in this study. Sixth, although gender differences were observed in this study, the underlying mechanisms for those influence factors remain unclear.

Regardless of those limitations, this study with a large sample size provided several contributions to current knowledge about suicidal ideation. 1) This study may be the first study conducted within a general population in an undeveloped urban area of China. 2) Detailed risk factors of suicidal ideation were investigated among a large number of populations. 3) The gender-specific differences were justified by using statistics analyses, which will be beneficial for the design and implementation of suicidal prevention strategies.

## Conclusion

In conclusion, suicide is an urgent public health issue in China, comprehensive suicide prevention strategies should be developed or strengthened in order to prevent suicide ideation in China. In the future, a longitudinal study is needed to confirm the casual relation between gender-specific influence factors and suicidal ideation, and more attention should be paid to gender difference in the prevention strategy for suicide.
